# Deep learning predicts HER2 status in invasive breast cancer from multimodal ultrasound and MRI

**DOI:** 10.17305/bb.2025.12475

**Published:** 2025-05-16

**Authors:** Yuhong Fan, Kaixiang Sun, Yao Xiao, Peng Zhong, Yun Meng, Yang Yang, Zhenwei Du, Jingqin Fang

**Affiliations:** 1 Department of Ultrasound Diagnosis, Daping Hospital, Army Medical University, Chongqing, China; 2 Jinfeng Laboratory, Chongqing, China; 3 Department of Pathology, Daping Hospital, Army Medical University, Chongqing, China; 4 Department of Medical Engineering, Daping Hospital, Army Medical University, Chongqing, China

**Keywords:** Breast neoplasms, ERBB2 protein, human, ultrasound, US, magnetic resonance image, MRI, deep learning, DL

## Abstract

The preoperative human epidermal growth factor receptor type 2 (HER2) status of breast cancer is typically determined by pathological examination of a core needle biopsy, which influences the efficacy of neoadjuvant chemotherapy (NAC). However, the highly heterogeneous nature of breast cancer and the limitations of needle aspiration biopsy increase the instability of pathological evaluation. The aim of this study was to predict HER2 status in preoperative breast cancer using deep learning (DL) models based on ultrasound (US) and magnetic resonance imaging (MRI). The study included women with invasive breast cancer who underwent US and MRI at our institution between January 2021 and July 2024. US images and dynamic contrast-enhanced T1-weighted MRI images were used to construct DL models (DL-US: the DL model based on US; DL-MRI: the model based on MRI; and DL-MRI&US: the combined model based on both MRI and US). All classifications were based on postoperative pathological evaluation. Receiver operating characteristic analysis and the DeLong test were used to compare the diagnostic performance of the DL models. In the test cohort, DL-US differentiated the HER2 status of breast cancer with an AUC of 0.842 (95% CI: 0.708–0.931), and sensitivity and specificity of 89.5% and 79.3%, respectively. DL-MRI achieved an AUC of 0.800 (95% CI: 0.660–0.902), with sensitivity and specificity of 78.9% and 79.3%, respectively. DL-MRI&US yielded an AUC of 0.898 (95% CI: 0.777–0.967), with sensitivity and specificity of 63.2% and 100.0%, respectively.

## Introduction

Human epidermal growth factor receptor type 2 (HER2) is one of the most important biomarkers in breast cancer [1]. The literature suggests that patients with HER2-positive invasive breast cancer are more likely to benefit from neoadjuvant chemotherapy (NAC) than those with HER2-negative disease [2, 3]. Therefore, accurate preoperative assessment of HER2 status is crucial for clinicians in developing an effective treatment plan. Preoperative evaluation of HER2 status typically relies on immunohistochemical (IHC) analysis of core needle biopsy samples. HER2-positive breast cancer is defined as an IHC score of 3+ in at least one tumor sample, or an IHC score of 2+ accompanied by a positive fluorescence in situ hybridization (FISH) test indicating gene amplification. Conversely, HER2-negative breast cancer is defined as an IHC score of 0 or 1+, or an IHC score of 2+ with a negative FISH result [2]. However, HER2-positive breast cancers are known to be highly heterogeneous, and core needle biopsies may not fully represent the HER2 status of the entire tumor [4]. Additionally, breast cancer is a progressive disease, and HER2 expression can vary within and between lesions over time [4, 5]. These factors contribute to uncertainty in HER2 status assessment. Ultrasonography (US) and magnetic resonance imaging (MRI) are the most commonly used imaging techniques in breast cancer diagnosis. Numerous studies have investigated the use of US- or MRI-derived parameters—such as radiomic and clinical features—to predict HER2 status [6–14]. These studies have demonstrated the potential value of imaging in HER2 status prediction. However, the extraction of conventional and radiomic features is often highly operator-dependent. Deep learning (DL) addresses this limitation by automatically extracting features from medical images using deep neural networks. To the best of our knowledge, there is limited published research on using DL to predict HER2 status in invasive breast cancer. Therefore, the aim of this study was to develop DL models based on US and MRI to predict HER2 status in invasive breast cancer.

## Materials and methods

### Patients

**Figure 1.**
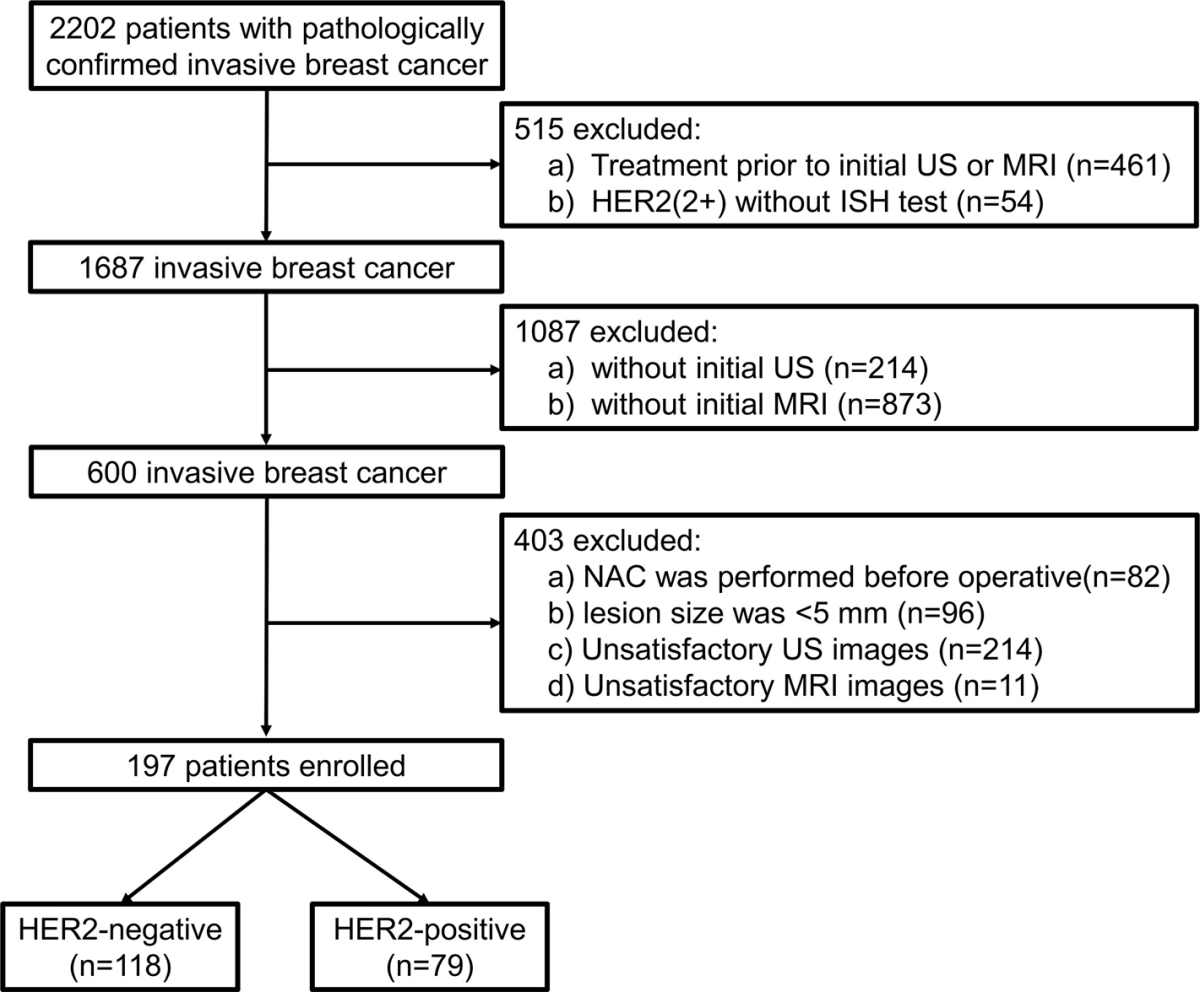
**Flowchart for the selection of participants.** MRI: Magnetic resonance imaging; US: Ultrasound; NAC: Neoadjuvant chemotherapy; HER2: Human epidermal growth factor receptor type 2.

This study consecutively included 197 patients with pathologically confirmed invasive breast cancer between January 2021 and July 2024. The inclusion criteria were as follows: (1) age >18 years; (2) pathologically confirmed invasive breast cancer with a clearly documented HER2 grade in the postoperative pathological report; and (3) both ultrasound (US) and MRI performed within a two-week interval. The exclusion criteria were: (1) NAC or core needle biopsy performed prior to US or MRI; (2) NAC performed before surgery; (3) poor image quality of US or MRI; (4) lesion size <5 mm; and (5) absence of enhancement in the final three-dimensional T1-weighted contrast-enhanced sequence (Figure 1).

### US and MRI imaging protocol

The DC8 US diagnostic system (Mindray Medical International Co., Ltd., Shenzhen, China), equipped with a 3–12 MHz linear-array transducer, was used for the imaging protocol, which included greyscale and color Doppler image acquisition in two orthogonal planes. US images of all breast masses in long-axis and short-axis views were stored in the Picture Archiving and Communication System (PACS) for subsequent image analysis. In accordance with the 2013 American College of Radiology (ACR) Breast Imaging Reporting and Data System (BI-RADS) lexicon, two radiologists extracted BI-RADS features from the US images—including shape, margin, orientation, echo pattern, posterior features, calcifications, vascularity, and axillary lymph nodes [16]. The radiologists were blinded to both the pathological findings and the MRI results. Any disagreements were resolved through consensus-based discussion. Longitudinal-section images of the breast masses were selected for DL and data analysis of the lesions. All patients underwent MRI examination using a 1.5 T scanner (Magnetom Aera, Siemens Healthcare, Erlangen, Germany) with an eight-channel dedicated breast phased-array coil. The scanning sequences and parameters were as follows: (a) axial T1-weighted imaging (T1WI): repetition time (TR) ═ 8.6 ms, echo time (TE) ═ 4.7 ms, field of view (FOV) ═ 360 mm × 360 mm, matrix ═ 384 × 384, slice thickness ═ 4.0 mm; (b) axial T2-weighted imaging with fat suppression (T2WIFS): TR ═ 5600 ms, TE ═ 57 ms, FOV ═ 340 mm × 340 mm, slice thickness ═ 4.0 mm; (c) axial dynamic contrast-enhanced T1WI (DCE-T1WI): TR ═ 4.62 ms, TE ═ 1.75 ms, FOV ═ 360 mm × 360 mm, slice thickness ═ 1.5 mm. DCE-T1WI was acquired using the TWIST-VIBE technique. A pre-contrast, single-phase T1WI scan (scan time: 90 s) was performed before contrast injection. A gadolinium-based contrast agent (Magnevist, Bayer Healthcare, Berlin, Germany) was then injected at a dose of 0.1 mmol/kg and a flow rate of 2.0 mL/s, followed by a 20 mL saline flush at the same rate. Six post-contrast phases were acquired consecutively without interval. Each scan lasted approximately 60.1 s with a slice thickness of 3 mm, and the total scan time was 6 min and 9 s. All images were assessed based on the ACR BI-RADS MRI lexicon [16]. MRI features included tumor shape (round/oval or irregular), margin (circumscribed or non-circumscribed), largest tumor size, number of lesions (single or multiple), intratumoral T2 hypersignal (present or absent), skin involvement (present or absent), nipple involvement (present or absent), internal enhancement pattern (homogeneous or heterogeneous), and presence or absence of non-mass enhancement.

### DL model of US: Based on the ConvNeXt V2 model

**Figure 2.**
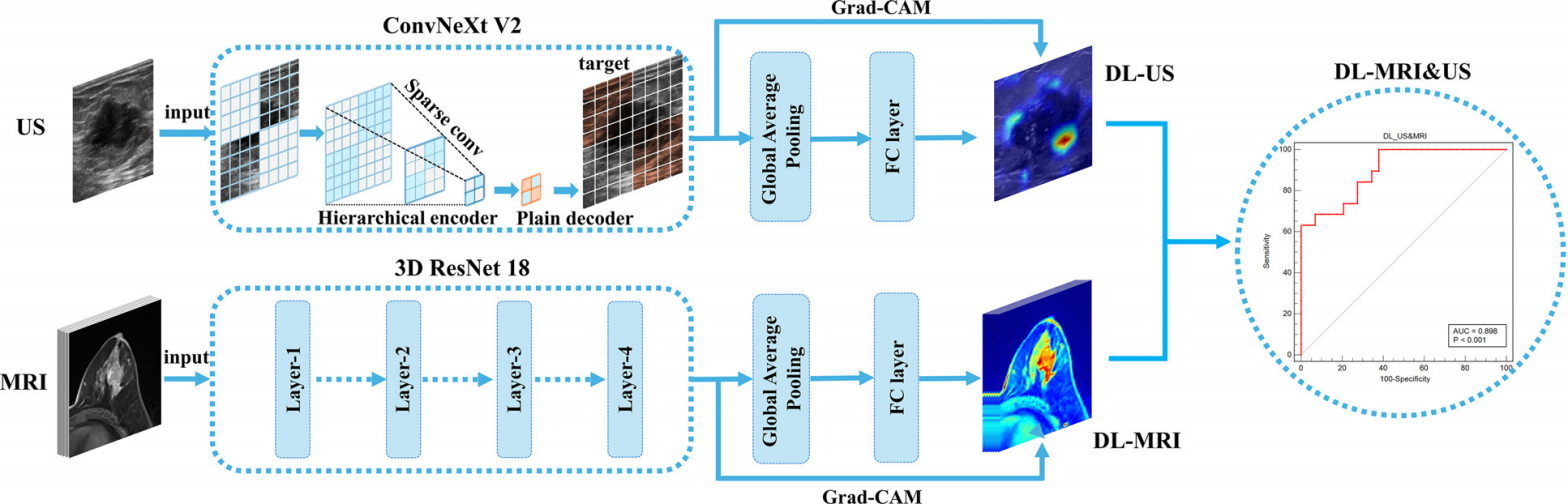
**The structure of deep learning model system.** MRI: Magnetic resonance imaging; US: Ultrasound; DL: Deep learning; Grad-CAM: Gradient-weighted class activation mapping.

The ConvNeXt V2 model leverages the strengths of convolutional neural networks and enhances feature extraction from US images by optimizing both the network structure and parameter configuration. It has been widely adopted for DL tasks involving US images. Clear grayscale US images of the longitudinal section of breast cancer are selected as inputs for the DL model. Clinicians first use ITK-SNAP to manually delineate the tumor region mask, identifying the initial tumor location. Given the typically small size of breast cancer lesions, the initial mask is extended outward by 50 pixels to create the final tumor mask. Based on this mask, the tumor and its surrounding region of interest (ROI) are extracted to provide effective input data for subsequent model training. The images are then normalized using Z-score normalization and resized to 256 × 256 pixels. Various data augmentation techniques—including random selection, flipping, scaling, panning, Gaussian noise addition, Gaussian blurring, and brightness/contrast adjustment—are applied during preprocessing. The preprocessed US images are then fed into the ConvNeXt V2 model for training (optimizer: AdamW; initial learning rate: 2e-5; weight decay: 5e-2; batch size: 16). Four-fold cross-validation is used for both training and evaluation. Additionally, dynamic loss scaling and Mixup data augmentation strategies are employed to further enhance the model’s generalization and classification performance (Figure 2). After 300 training epochs, the model’s optimal weights are determined based on accuracy, recall, AUC, and F1 score. These optimal weights are then used to generate predicted probabilities (ranging from 0 to 1) for each category in the test cohort.

### DL model of MRI: Based on the 3D Resnet model

The 3D ResNet18 model features a relatively simple architecture and a moderate number of parameters compared to other 3D models, such as EfficientNet, ResNet, and ConvNeXt V2. Its design makes it more likely to converge when processing low-quality MRI images. Additionally, the model’s residual connections help mitigate the vanishing gradient problem, enabling it to better learn image features during training and reducing the risk of overfitting. As a result, 3D ResNet18 is widely used in DL applications involving MRI images. In this study, the latest three-dimensional T1-weighted contrast-enhanced MRI sequences of breast cancer were used as input for DL. Physicians manually delineated tumor masks layer by layer using ITK-SNAP, then expanded each initial mask outward by 50 pixels to form the final tumor mask. Based on this mask, both the tumor and its surrounding ROI were extracted to generate effective input data for model training. The images were normalized using Z-score normalization and resized to 96 × 96 × 96 to meet the input requirements of the 3D convolutional network. A variety of data augmentation techniques—including random selection, flipping, scaling, panning, Gaussian noise, Gaussian blur, and brightness/contrast adjustments—were applied during preprocessing. The preprocessed breast cancer MRI images were then fed into the 3D ResNet18 model for training, using the AdamW optimizer (initial learning rate: 4e-6; weight decay: 1e-2; batch size: 16). Four-fold cross-validation was employed for both training and evaluation. The 3D ResNet18 efficiently extracts multi-scale features via its residual connections and utilizes 3D convolutions to capture the textural and structural characteristics of tumors in three-dimensional space, enabling accurate identification of HER2 status (see Figure 2). The model was trained for 300 epochs, and the optimal weights were selected based on metrics, including accuracy, recall, AUC, and F1 score. Final predictions for each category in the test cohort were output as probability scores (range: 0–1) using the best-performing model weights. Stratified sampling was used to divide the dataset for cross-validation, ensuring a consistent distribution of HER2 categories in both training and test sets to address class imbalance. Details of all Python libraries used are provided in Table S1. The full source code for the DL models is available at: https://github.com/sun-kx/Ultrasound_and_MRI_predict_HER2_status_in_invasive_breast_cancer.

### DL models for US and MRI

To further evaluate whether DL-US&MRI offers superior performance in distinguishing breast cancer HER2 status, we developed the DL-US&MRI model using logistic regression, incorporating the predictive values of DL-US and DL MRI model (DL-MRI) in the test cohort (Figure 2).

### Pathological evaluation

The diagnosis of invasive breast cancer in all patients was confirmed by postoperative pathological evaluation. Breast cancer IHC types were determined based on ER, PR, HER2 receptor status, and Ki-67 levels [17]. ER- and PR-positive status was defined as ≥1% positively stained tumor cells on IHC [18]. HER2 positivity was defined as IHC 3+, or IHC 2+ with amplification confirmed by FISH. HER2 negativity was defined as IHC 0, 1+, or 2+ with a negative FISH result [1, 19]. The threshold level for Ki-67 was set at 14% [17]. Molecular subtypes were categorized as hormone receptor (HR) positive or HR negative. HR positivity was defined as expression of ER and/or PR in more than 10% of invasive cancer cells [20].

### Ethical statement

This single-center study was approved by the institutional ethics review board (Ratification No: 2024(242)). Informed consent was waived due to the retrospective nature of the study. The study complied with the tenets of the Declaration of Helsinki and adhered to the Standards for Reporting Diagnostic Accuracy [15].

### Statistical analysis

MedCalc Statistical Software version 20.010 (MedCalc Software bvba, Ostend, Belgium) and Python 3.10 (Python Software Foundation, Beaverton, USA) were used for data analysis in this study. The normality of continuous variables was assessed using the Shapiro–Wilk test. Variables following a normal distribution were expressed as mean ± standard deviation, while those not normally distributed were presented as median (quartiles). The independent samples *t*-test was used to compare normally distributed data between two groups, whereas the Mann–Whitney *U* test was applied for non-normally distributed data. Categorical data were reported as frequencies and percentages, and group differences were assessed using the chi-squared test or Fisher’s exact test, as appropriate. *P* values for multiple comparisons were adjusted using Bonferroni’s correction. The area under the receiver operating characteristic (ROC) curve (AUC) was used to evaluate model performance. A *P* value of <0.05 was considered statistically significant. All Python packages and libraries used in the study are listed in Table S1.

## Results

### Baseline characteristics

This single-center study enrolled 197 patients with invasive breast cancer who met the inclusion and exclusion criteria. Among them, 118 were HER2-negative and 79 were HER2-positive. Stratified random sampling was used to divide the data into a training cohort (149 cases) and a test cohort (48 cases). Table 1 summarizes the baseline characteristics of the patients, while Tables 2 and 3 present the US and MRI characteristics, respectively. There were no significant differences in baseline, US, or MRI characteristics between the training and test cohorts.

**Table 1:** Clinical and pathological findings of the study sample

**Variables**	**Train (*n* ═ 149)**	**Test (*n* ═ 48)**	***P***
Age, median (Q1, Q3)	51 (46, 58)	50 (46, 57)	0.704
*Location, n(%)*			0.761
Left	78 (52)	27 (56)	
Right	71 (48)	21 (44)	
*Ki 67, n(%)*			0.823
Low	42 (28)	15 (31)	
High	107 (72)	33 (69)	
*ER, n(%)*			0.566
Negative	49 (33)	13 (27)	
Positive	100 (67)	35 (73)	
*PR, n(%)*			0.554
Negative	62 (42)	17 (35)	
Positive	87 (58)	31 (65)	
*HER2 status, n(%)*			0.933
Negative	89 (60)	29 (60)	
Positive	60 (40)	19 (40)	
*HR status, n(%)*			0.811
Negative	51 (34)	18 (38)	
Positive	98 (66)	30 (62)	
*Luminal, n(%)*			0.966
Negative	46 (31)	14 (29)	
Positive	103 (69)	34 (71)	
*HER2 positive (non luminal), n(%)*			0.993
Negative	117 (79)	37 (77)	
Positive	32 (21)	11 (23)	
*Triple negative (ductal), n(%)*			0.768
Negative	135 (91)	45 (94)	
Positive	14 (9)	3 (6)	

HR: Hormone receptor; HER2: Human epidermal growth factor receptor type 2; ER: Estrogen receptor; PR: Progesterone receptor.

**Table 2:** US findings of the study sample

**Variables**	**Train (*n* ═ 149)**	**Test (*n* ═ 48)**	***P***
*Shape, n(%)*			0.684
Round/oval	40 (27)	15 (31)	
Irregular	109 (73)	33 (69)	
*Orientation, n(%)*			0.991
Parallel	86 (58)	27 (56)	
Not parallel	63 (42)	21 (44)	
*Margin, n(%)*			0.502
Circumscribed	20 (13)	9 (19)	
Not circumscribed	129 (87)	39 (81)	
*Echo pattern, n(%)*			0.177
Hypoechoic	134 (90)	38 (79)	
Heterogeneous	7 (5)	5 (10)	
Complex cystic and solid	5 (3)	3 (6)	
Isoechoic	3 (2)	2 (4)	
*Posterior features, n(%)*			0.478
Enhancement	24 (16)	9 (19)	
No posterior features	73 (49)	24 (50)	
Shadowing	44 (30)	15 (31)	
Combined pattern	8 (5)	0 (0)	
*Calcifications, n(%)*			0.658
Absent	57 (38)	16 (33)	
Present	92 (62)	32 (67)	
*Vascularity, n(%)*			0.633
Internal vascularity	99 (66)	31 (65)	
Vessels in rim	7 (5)	4 (8)	
Absent	43 (29)	13 (27)	
*Lymph nodes axillary, n(%)*			0.534
Absent	93 (62)	33 (69)	
Present	56 (38)	15 (31)	

US: Ultrasound.

**Table 3:** MRI findings of the study sample

**Variables**	**Train (*n* ═ 149)**	**Test (*n* ═ 48)**	***P***
*Shape, n(%)*			0.835
Round/oval	30 (20)	11 (23)	
Irregular	119 (80)	37 (77)	
*Tumor margins, n(%)*			0.836
Circumscribed	73 (49)	25 (52)	
Not circumscribed	76 (51)	23 (48)	
Largest tumor size, median (Q1, Q3)	23 (17, 29)	21 (16.75, 26)	0.243
*No of lesions, n(%)*			0.791
Single	138 (93)	45 (94)	
Multiple	11 (7)	3 (6)	
*MRI intratumoral T2 hypersignal, n(%)*			0.146
Absent	70 (47)	29 (60)	
Present	79 (53)	19 (40)	
*Skin involvement, n(%)*			0.132
Absent	129 (87)	46 (96)	
Present	20 (13)	2 (4)	
*Nipple involvement, n(%)*			0.568
Absent	130 (87)	44 (92)	
Present	19 (13)	4 (8)	
*Enhancement pattern, n(%)*			0.536
Homogenous	78 (52)	22 (46)	
Heterogenous	71 (48)	26 (54)	
*Nonmass enhancement, n(%)*			0.392
Absent	125 (84)	37 (77)	
Present	24 (16)	11 (23)	

MRI: Magnetic resonance imaging.

### Performance of DL-US

**Figure 3.**
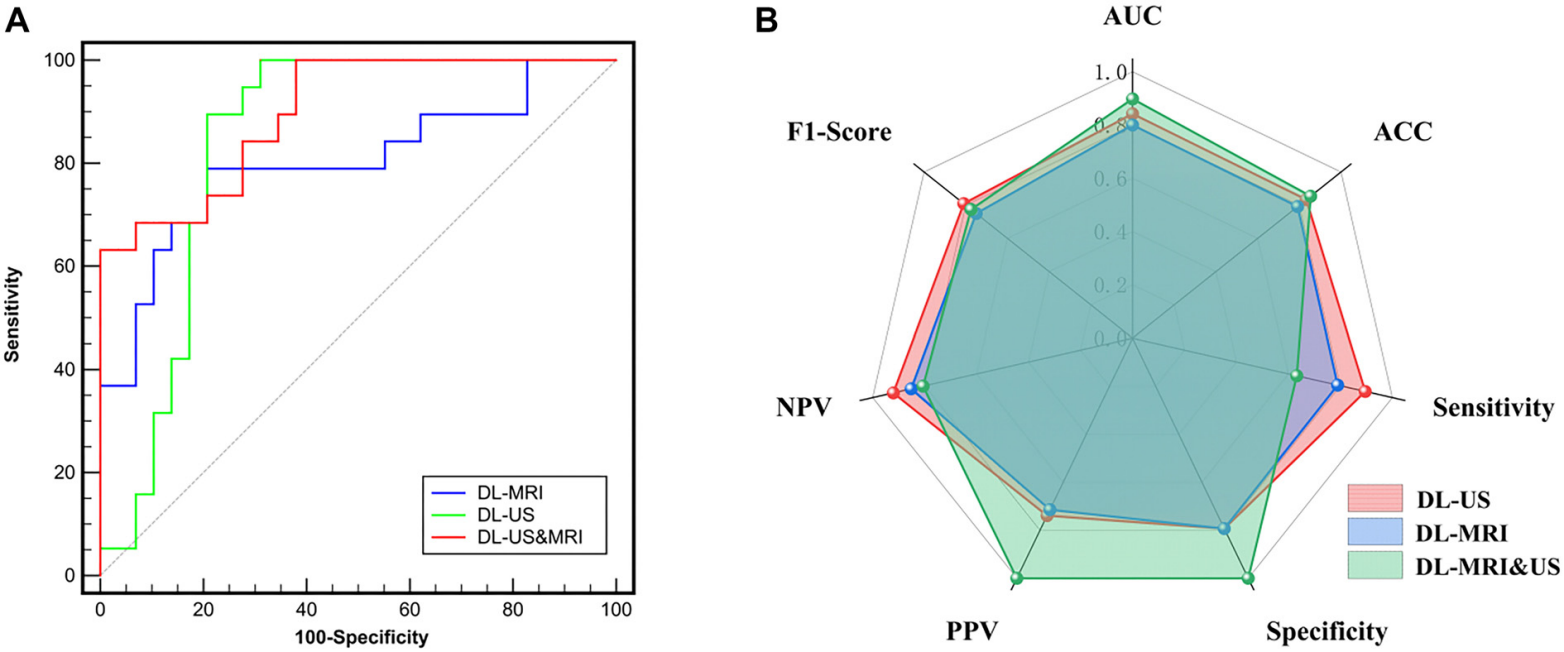
**The performance of deep learning models.** (A) The area under the receiver operating characteristic curve; (B) Parameters of three deep learning models. MRI: Magnetic resonance imaging; US: Ultrasound; DL: Deep learning; NPV: Negative predictive value; PPV: Positive predictive value; AUC: Area under the curve; ACC: Accuracy.

In the test cohort, DL-US was used to differentiate HER2 status in breast cancer. The model achieved an AUC of 0.842 (95% CI: 0.708–0.931), with an accuracy of 0.833, sensitivity of 89.5%, specificity of 79.3%, PPV of 0.739, NPV of 0.920, and an F1 score of 0.810 (Figure 3).

### Performance of DL-MRI

In the test cohort, DL-MRI was used to differentiate HER2 status in breast cancer, achieving an AUC of 0.800 (95% CI: 0.660–0.902). The DL-MRI also demonstrated an accuracy of 0.791, with a sensitivity of 78.90%, specificity of 79.30%, PPV of 0.714, NPV of 0.852, and an F1 score of 0.750 (Figure 3).

### Performance of DL-US&MRI

In the test cohort, the DL-US&MRI model achieved an AUC of 0.898 (95% CI: 0.777–0.967) for differentiating HER2 status in breast cancer. The model also demonstrated an accuracy of 0.854, sensitivity of 63.20%, specificity of 100.00%, PPV of 1.000, NPV of 0.806, and an F1 score of 0.775. Model performance comparisons were conducted using the DeLong test. Although DL-US&MRI outperformed both DL-US and DL-MRI, the differences were not statistically significant (*P* ═ 0.2746, AUC: 0.898 vs 0.842; *P* ═ 0.0538, AUC: 0.898 vs 0.800). DL-US also showed higher performance than DL-MRI, but this difference was likewise not statistically significant (*P* ═ 0.6595, AUC: 0.842 vs 0.800) (Figure 3).

### Interpretability of DL-MRI and DL-US

**Figure 4.**
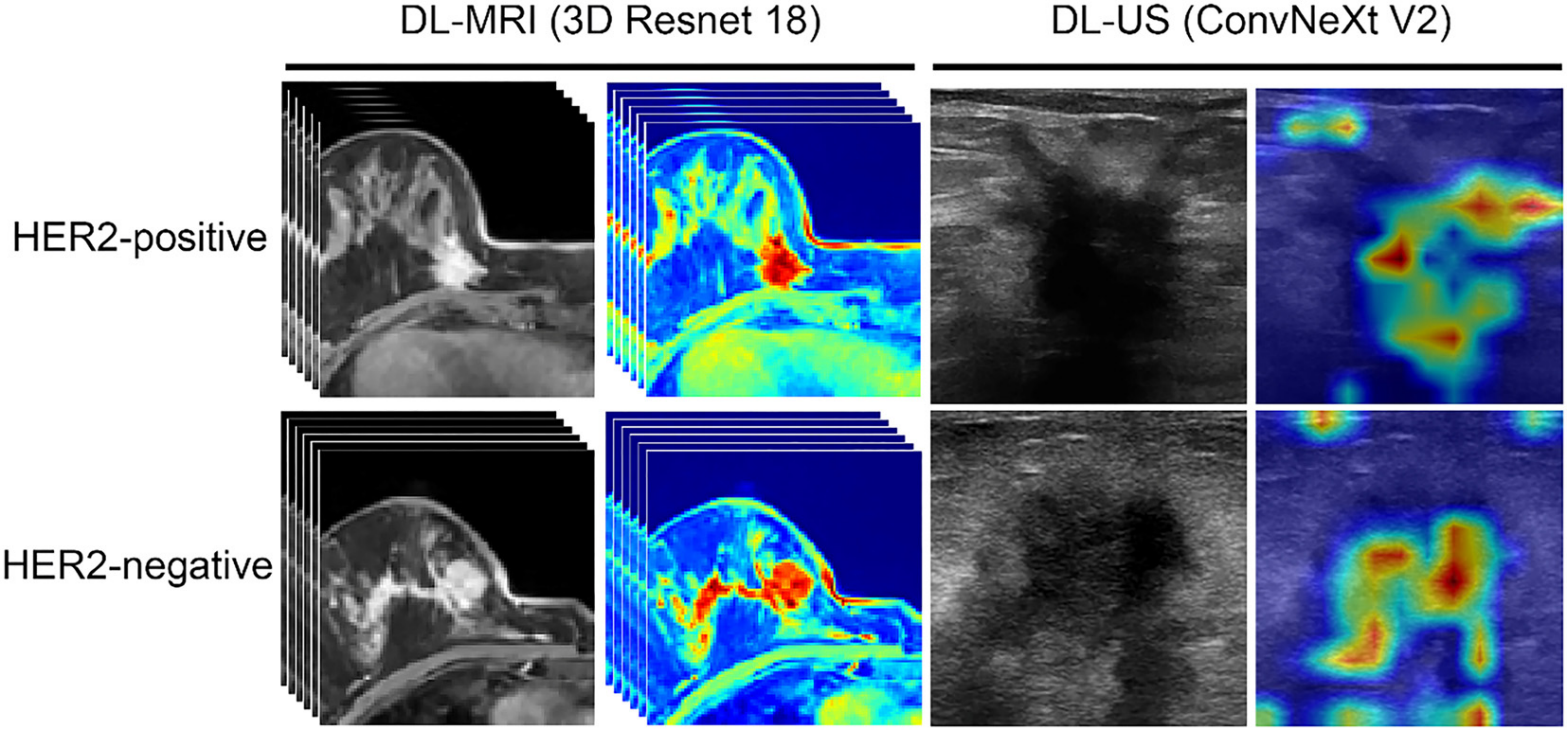
**Grad-CAM visualization: Visualization of DL-MRI and DL-US.** MRI: Magnetic resonance imaging; US: Ultrasound; DL: Deep learning; Grad-CAM: Gradient-weighted class activation mapping; HER2: Human epidermal growth factor receptor type 2.

To explore the interpretability of DL-MRI and DL-US, we use gradient-weighted class activation mapping (Grad-CAM) to visualize the regions of greatest interest identified by each model, as shown in Figure 4.

## Discussion

HR status in breast cancer affects the efficacy of NAC, with HER2 status playing a critical role [2, 3]. Pathological biopsy remains the gold standard for determining HER2 status preoperatively; however, due to the heterogeneity of breast cancer and the sampling limitations of core needle biopsy, there is a need for methods that can comprehensively assess HER2 status before surgery [4, 5]. US and MRI are two of the most widely used imaging modalities in breast cancer and have demonstrated significant value in evaluating HER2 status. Previous studies primarily employed machine learning models based on radiomics, BI-RADS lexicon features, and clinical data—11 using MRI [10–14, 21–26] and four using US [6–9]. In this study, DL models showed strong performance in predicting HER2 status in invasive breast cancer, with AUC values of 0.845 for DL-US, 0.800 for DL-MRI, and 0.898 for the combined DL-MRI&US model. MRI, with its superior soft tissue contrast, has emerged as a key noninvasive tool for assessing HER2 status, capturing tumor spatial heterogeneity. Performance of MRI-based models varies depending on classification granularity and methodological rigor. For binary classification (HER2-positive vs HER2-negative), conventional radiomics models [11, 13, 21, 22, 24, 25] reported external AUCs ranging from 0.68 to 0.84. The study by Xu et al. [25] achieved a higher AUC of 0.945 by incorporating clinical features, such as Ki-67 and histologic grade. In contrast, our 3D DL-MRI, which excluded clinical variables, achieved an AUC of 0.800—bridging the performance gap between pure radiomics and clinical-radiomics hybrids. For HER2 low/zero subtyping [10, 23], the nomogram by Yin et al. achieved an external AUC of 0.886 [23], which was comparable to our DL-US&MRI model (AUC: 0.898), despite differing in methodology (2D radiomics vs 3D DL). Importantly, our cross-modal fusion model (DL-MRI&US) outperformed stand-alone MRI (ΔAUC: +0.098), emphasizing the complementary strengths of multimodal imaging. Multiclass classification frameworks, such as the study by Zhang et al. distinguishing HER2-zero, -low, and -positive, reported AUCs of 0.80–0.85 [12], aligning with our binary performance but highlighting a trade-off between classification granularity and model generalizability. Persistent limitations—including single-center data, undersampling of HER2-low/null cases, and reliance on 2D spatial features [11, 23, 25]—were partially addressed through our use of 3D segmentation. Nonetheless, these issues call for further validation in multicenter studies. Future work should focus on integrating clinical and genomic data with 3D DL architectures to resolve molecular imaging discordance and further advance precision oncology.

US, as the most commonly used imaging technique for breast assessment, also plays an important role in evaluating HER2 status in invasive breast cancer. Our DL-US model, developed to distinguish between HER2-positive and HER2-negative breast cancers, demonstrated robust performance in our study cohort (AUC: 0.842; sensitivity: 89.5%; specificity: 79.3%). This outcome definition aligns with the studies by Ferre et al. (HER2+ vs HER2-, AUC: 0.778) [6] and Cui et al. (HER2+ vs HER2-, AUC: 0.844) [7], but differs from those by Zhang et al. (HER2-low vs HER2-zero, AUC: 0.84) [8] and Zhou et al. (HER2+ non-luminal vs other, AUC: 0.725) [9], where subclass-specific classifications may limit clinical generalizability. Methodologically, the DL-US model outperformed conventional radiomics approaches that rely on hand-crafted features (e.g., wavelet-based GLSZM/GLRLM) and clinical-US hybrids [6–9], likely due to its capacity to autonomously extract discriminative hierarchical patterns from raw image data. While logistic regression dominated prior studies (AUCs: 0.778–0.844) [6, 7], its dependence on manual feature engineering limits adaptability to HER2 heterogeneity—an issue DL-US overcomes via end-to-end learning. Despite its superior performance metrics, the “black box” nature of the DL model contrasts with the interpretability of radiomics, underscoring a trade-off between predictive accuracy and biological insight. Overall, these findings support DL-US as a promising non-invasive tool for HER2 status prediction, although multicenter prospective validation is essential to confirm clinical utility. In the current study, we developed a joint DL model (DL-MRI&US) that integrates US and MRI data to predict HER2 status in invasive breast cancer, achieving an AUC of 0.898. To our knowledge, this is the first published model to combine these imaging modalities for HER2 prediction, and it outperformed all previously reported approaches. While our findings underscore the potential of imaging biomarkers, it is important to note that preoperative prognostic evaluation in breast cancer should also incorporate clinical parameters. Emerging evidence links preoperative biological markers—including the neutrophil-to-eosinophil ratio (NER), indicators of inflammation and metabolic syndrome, and the Cachexia Index (CXI)—with breast cancer outcomes [27–29]. Future research that integrates imaging biomarkers with clinical parameters through multimodal fusion models may yield improved predictive performance for assessing NAC efficacy. Our results show that the multimodal DL-US&MRI model achieved a clinically meaningful 9.8% absolute improvement in AUC over DL-MRI alone (*P* ═ 0.0538), an effect size consistent with thresholds for clinically actionable advances. Although this difference did not reach conventional statistical significance—likely due to cohort size limitations—the magnitude of improvement suggests potential clinical relevance, particularly given the model’s 100% specificity, which may reduce false-positive referrals compared to DL-US (specificity: 75.6%). However, the lower sensitivity of the multimodal model (63.2%) positions it more appropriately as a confirmatory tool within a staged diagnostic workflow rather than as a stand-alone screening method. In contrast, the higher sensitivity of the US-only model may be more suitable for initial screening, especially in resource-limited settings. Both applications highlight the need for larger validation cohorts to address statistical uncertainties—particularly regarding the borderline significance of the multimodal model—and to clarify its potential benefits in reducing diagnostic errors, patient anxiety, and healthcare costs through improved specificity. The complementary strengths of the DL-US and DL-US&MRI models suggest promise for coordinated implementation across different diagnostic pathways.

This study has several limitations. First, the small sample size, due to strict inclusion and exclusion criteria, precluded subgroup analyses distinguishing between HER2-zero, HER2-low, and HER2-high categories. Second, the potential additive value of radiomic features for predicting HER2 status remains unexplored within our DL framework. Third, the lack of external validation limits the assessment of the model’s generalizability. Future large-scale, multicenter studies with diverse cohorts are needed to address these limitations and meet emerging clinical needs. Notably, recent evidence suggests that HER2-low breast cancers (defined as IHC 1+ or FISH-negative IHC 2+) may benefit from trastuzumab deruxtecan [30], underscoring the clinical importance of accurate HER2-low identification. Therefore, such studies should aim to validate imaging-based models while refining HER2-low detection capabilities to enhance prognostic stratification.

## Conclusion

In conclusion, DL models based on US and MRI demonstrate excellent performance in predicting HER2 status in invasive breast cancer. Moreover, combining both modalities can enhance the predictive accuracy of these models.

## Supplemental data

**Table S1:** Python packages and libraries used in this study

**Library name**	**Version**	**Developer/Company (Location)**
PyTorch	2.0	Meta AI (Menlo Park, USA)
Torchvision	0.15	Meta AI (Menlo Park, USA)
OpenMMLab	1.0.0	Shanghai AI Laboratory (Shanghai, China)
MMCV	2.0.0	Shanghai AI Laboratory (Shanghai, China)
MONAI	1.2.0	Project MONAI (USA)
Scikit-learn	1.3	Inria (Paris, France)
XGBoost	1.7	DMLC (USA)
OpenCV	4.7	OpenCV.org (Palo Alto, USA)
Matplotlib	3.7	Matplotlib Development Team (USA)
Seaborn	0.12	Michael Waskom (USA)
SimpleITK	2.3.0	Kitware Inc. (Clifton Park, USA)
NumPy	1.24	NumPy Developers (USA)
Pandas	1.5	Pandas Development Team (USA)
h5py	3.8	HDF Group (Champaign, USA)
Tqdm	4.65	Noam Yorav-Raphael (USA)
Ubuntu OS	22.04	Canonical Ltd. (London, UK)

For full reproducibility, the code and environment configuration are publicly available in our GitHub repository: https://github.com/sun-kx/Ultrasound_and_MRI_predict_HER2_status_in_invasive_breast_cancer.

## Data Availability

All data generated and analyzed during this study are included in this article.
